# Safety and Efficacy of Intravenous Immune Globulin 10% (BIVIGAM^®^) in Children with Primary Immune Deficiency

**DOI:** 10.1007/s10875-025-01891-1

**Published:** 2025-06-11

**Authors:** Isaac Melamed, Jolan E. Walter, Oral Alpan, BIVI 994 Authors, Devi Jhaveri, Alan Koterba, Rebecca Avila, Miranda Anaya, Marie-Chantale Simard, Wei Du, Jennifer W. Leiding

**Affiliations:** 1 IMMUNOe International Research, Centennial, CO USA; 2 Department of Pediatrics, Division of Allergy & Immunology, University of South Florida, Tampa, FL USA; 3 Department of Pediatrics, Division of Allergy & Immunology, Johns Hopkins All Children’s Hospital, St. Petersburg, Florida, USA; 4 Jeffrey Modell Diagnostic and Research Center for Primary Immunodeficiencies, Johns Hopkins All Children’s Hospital, St. Petersburg, Florida, USA; 5 Lysosomal Rare Disorders Research and Treatment Center, Fairfax, VA USA; 6 Department of Pediatrics, Division of Allergy and Immunology, Johns Hopkins University, Baltimore, MD USA; 7 Cancer and Blood Disorders Institute, Johns Hopkins All Children’s Hospital, St. Petersburg, Florida, USA; 8 Institute for Clinical and Translational Research, Johns Hopkins All Children’s Hospital, St. Petersburg, Florida, USA

**Keywords:** Immune globulin replacement therapy, Intravenous immune globulin, Pediatric population, Clinical trial, Safety, Efficacy

Up to 40% of patients with primary immune deficiency (PID) are diagnosed under the age of 18 [1] and younger patients are particularly vulnerable [2]. Immune globulin replacement is a standard therapy for PID [3]. However, little was known about the safety and efficacy of intravenous immune globulin IVIG 10% (BIVIGAM^®^) in children (e.g., only ten participants between 6 and 17 years of age were treated in the pivotal phase 3 trial [4] due to the difficulty of enrolling pediatric patients). Here we aimed to assess the safety, efficacy, and pharmacokinetics of BIVIGAM^®^ in children and adolescents with PID.

This phase 4, multi-center, prospective, open-label, single-arm clinical trial (NCT03164967) included participants with a documented PID diagnosis who had received intravenous immune globulin infusions every 3 or 4 weeks at a steady dose for ≥ 3 months prior to study entry and who maintained an immune globulin G (IgG) trough level of at least 500 mg/dL. Participants received BIVIGAM^®^ at the dose of 300–800 mg/kg administered intravenously every 3 or 4 weeks for 5 months; they were observed for 6 months. The study was part of a post-marketing requirement by the US Food and Drug Administration (FDA) [5]. The primary objective was safety; efficacy and pharmacokinetics were secondary objectives. All participants who received at least one dose of BIVIGAM^®^ and had at least one post-dosing follow-up visit were included in the safety/efficacy analysis set. See Online Resource: Supplementary Methods for further details.

Eighteen participants were enrolled, of whom 16 (n = 16 male, n = 13 White and non-Hispanic) received BIVIGAM^®^ every 3 weeks (n = 8) or 4 weeks (n = 8) and completed the study (Online Resource: Figure S1,Table S1). Ages ranged between 3 and 16 years (mean: 10.3 years): Three participants were 2– < 6 years old, five were 6– < 12 years old, and eight were 12–16 years old. Overall, the most common PID diagnosis was hypogammaglobulinemia (n = 8) followed by common variable immunodeficiency (n = 4), combined immunodeficiency (n = 2), Bruton's agammaglobulinemia (n = 1), and selective polysaccharide antibody deficiency (n = 1).

Participants in the safety analysis set received seven (3-week infusion regimen group) or five (4-week infusion regimen group) infusions each. Of the 96 total infusions, 45 were administered at ≥ 500 mg/kg and 51 were administered at < 500 mg/kg. Average doses ranged 349.8–1077.5 mg/kg in the 3-week infusion regimen group and 312.0–693.4 mg/kg in the 4-week infusion regimen group. Exposure length ranged 16.3–24.1 weeks in the 3-week infusion regimen group and 15.1–20.3 weeks in the 4-week infusion regimen group.

During the study, most participants (n = 13) experienced at least one treatment emergent adverse event (TEAE) (Table 1). Overall, 74 TEAEs were reported (average: 5.7 events per participant). Eight participants in the 3-week infusion regimen group experienced 62 TEAEs (average: 7.8 events per participant), and five participants in the 4-week infusion regimen group experienced 12 TEAEs (average: 2.4 events per participant). Events reported in ≥ 10% of participants included headache, upper respiratory infection, bronchitis, cough, fatigue, influenza, nasal congestion, oropharyngeal pain, pyrexia, and sinus congestion.

**Table 1:** Summary of adverse events

	3-week regimen (N = 8)		4-week regimen (N = 8)		Total (N = 16)	
	Total events	Participants n (%)*	Total events	Participants n (%)*	Total events	Participants n (%)*
Any adverse event	69	8 (100)	17	5 (63)	86	13 (81)
Any TEAE^†^	62	8 (100)	12	5 (63)	74	13 (81)
Infections and infestations	16	6 (75)	1	1 (13)	17	7 (44)
Upper respiratory tract infection	2	2 (25)	1	1 (13)	3	3 (19)
Bronchitis	3	2 (25)	0	0	3	2 (13)
Influenza	2	2 (25)	0	0	2	2 (13)
Respiratory, thoracic, and mediastinal disorders	12	4 (50)	3	3 (38)	15	7 (44)
Sinus congestion	3	1 (13)	1	1 (13)	4	2 (13)
Nasal congestion	2	1 (13)	1	1 (13)	3	2 (13)
Oropharyngeal pain	3	2 (25)	0	0	3	2 (13)
Cough	1	1 (13)	1	1 (13)	2	2 (13)
Nervous system disorders	12	4 (50)	4	2 (25)	16	6 (38)
Headache	5	3 (38)	3	2 (25)	8	5 (31)
General disorders and administration site conditions	5	2 (25)	3	2 (25)	8	4 (25)
Fatigue	4	1 (13)	1	1 (13)	5	2 (13)
Pyrexia	1	1 (13)	1	1 (13)	2	2 (13)
Gastrointestinal disorders	6	4 (50)	0	0	6	4 (25)
Injury, poisoning, and procedural complications	4	3 (38)	0	0	4	3 (19)
Metabolism and nutrition disorders	2	2 (25)	0	0	2	2 (13)
Blood and lymphatic system disorders	1	1 (13)	0	0	1	1 (6)
Immune system disorders	1	1 (13)	0	0	1	1 (6)
Musculoskeletal and connective tissue disorders	1	1 (13)	0	0	1	1 (6)
Psychiatric disorders	1	1 (13)	0	0	1	1 (6)
Skin and subcutaneous tissue disorders	1	1 (13)	0	0	1	1 (6)
Vascular disorders	0	0	1	1 (13)	1	1 (6)
Treatment-related adverse event	5	2 (25)	2	1 (13)	7	3 (19)
Procedural headache	3	2 (25)	0	0	3	2 (13)
Fatigue	2	1 (13)	0	0	2	1 (6)
Headache	0	0	2	1 (13)	2	1 (6)
Discontinuations due to adverse event	0	0	0	0	0	0
Serious adverse events leading to death	0	0	0	0	0	0
TAAE within 1 h of infusion^‡^	7	3 (38)	0	0	7	3 (19)
TAAE within 24 h of infusion^‡^	7	3 (38)	0	0	7	3 (19)
TAAE within 72 h of infusion^‡^	7	3 (38)	2	1 (13)	9	4 (25)
Adverse events of special interest^§^	0	0	0	0	0	0
Adverse infusion reaction	5	2 (25)	2	1 (13)	7	3 (19)
Infusion site reaction	0	0	0	0	0	0

^*^Counted once per preferred term/system organ class. ^†^Onset on/after first infusion. ^‡^Onset from infusion start until *x* h after infusion end; inclusive of previous time marks. ^§^Medical concerns specific to study drug requiring monitoring and investigation. TAAE = temporally-associated adverse event; TEAE = treatment-emergent adverse event

Three participants experienced seven TEAEs that were assessed as related to the study drug, including procedural headache (two participants experiencing three events), fatigue, and headache (each with one participant experiencing two events). Treatment-related procedural headache and fatigue were only reported with the 3-week regimen, whereas treatment-related headaches were only reported with the 4-week regimen.

Overall, most reported TEAEs were moderate (41/74 events) or mild (32/74 events) in intensity (Online Resource: Table S2), and only one TEAE (hemiparesis reported as left-side weakness in a 15-year-old male in the 3-week infusion regimen group) was deemed severe during the study. This event was reported as a serious adverse event (SAE), but it resolved on the following day, did not require a dose change, and was assessed as not related to the study drug. Alternative etiology was reported with possible psychological component, including conversion disorder or neuro-immune presentation related to common variable immune deficiency. No severe TEAEs/SAEs occurred in the 4-week infusion regimen group. Across the sample, the highest TEAE intensity was mild in four participants, moderate in eight participants, and severe in one participant. No TEAEs leading to discontinuation or death, no adverse events of special interest, and no infusion site reactions occurred during the study period.

The primary efficacy endpoint was the incidence of acute serious bacterial infections (SBIs). No acute SBIs occurred during study (Online Resource: Table S3**)**. A total of 17 infections occurred in seven participants and six participants had infections requiring antibiotics for a mean of 16.5 days (range, 1–35 days), but none required hospitalization (Online Resource: Table S4).

In all 10 participants in whom pharmacokinetics were evaluated (n = 3 in the 3-week regimen group and n = 7 in the 4-week regimen group), trough IgG levels were ≥ 500 mg/dL. There were no apparent differences in the total IgG or subclass concentrations before first and last infusion, regardless of age and frequency of dosing; patients receiving infusions every 3 weeks may have reached higher IgG concentrations than those treated every 4 weeks (Online Resource: Tables S5–S6).

The study had some limitations, including sample size, no female and few younger participants enrolled. Interpretation of the results; in particular, pharmacokinetic results should be regarded as qualitative. Longer surveillance could be required to further assess efficacy and safety in this population.

In conclusion, treatment with BIVIGAM^®^ in pediatric patients with PID was safe, effective, and met all pre-specified endpoints in children and adolescents with PID aged 3–16 years. Five months of treatment was well tolerated and the safety profile was generally consistent with the pivotal study [4], with no acute SBIs reported during the 6-month observation period. The dosing regimen (300–800 mg/kg every 3 or 4 weeks) appears to be appropriate to attain sufficient trough levels in this patient population. Study results support the use of BIVIGAM^®^ in children and adolescents with PID. Consequently, BIVIGAM^®^ has received FDA approval for use in patients aged ≥ 2 years [5].

## Supplementary Information

Below is the link to the electronic supplementary material. 

ESM1 (DOC 106 KB)

## Data Availability

The data supporting the findings of this study are available from the corresponding author upon reasonable request.
