# Supplementary material for: Safety and Efficacy of Intravenous Immune Globulin 10% (BIVIGAM®) in Children with Primary Immune Deficiency
Source: J Clin Immunol. 2025 Jun 11;45(1):105. doi: 10.1007/s10875-025-01891-1 (PMC12158822; doi:10.1007/s10875-025-01891-1)
Supplement: Supplementary file 1 — (DOC 106 KB) [file 10875_2025_1891_MOESM1_ESM.doc]

*Journal of Clinical Immunology*

Supplementary Information for:

**Intravenous Immune Globulin 10% (BIVIGAM^®^) in Children with Primary Immune Deficiency**

Isaac Melamed, MD*; Jolan E. Walter, MD, PhD^†,‡,§^; Oral Alpan, MD^‖^; BIVI 994 Authors^¶^; Jennifer W. Leiding, MD^‡‡,§§,‖‖^

* IMMUNOe International Research, Centennial, Colorado, USA

^†^ Department of Pediatrics, Division of Allergy & Immunology, University of South Florida, Tampa, Florida, USA

^‡^ Department of Pediatrics, Division of Allergy & Immunology, Johns Hopkins All Children’s Hospital, St. Petersburg, Florida, USA

^§^ Jeffrey Modell Diagnostic and Research Center for Primary Immunodeficiencies, Johns Hopkins All Children’s Hospital, St. Petersburg, Florida, USA

**^‖^** Lysosomal Rare Disorders Research and Treatment Center, Fairfax, Virginia, USA

^¶^ Author Group: Devi Jhaveri, DO (Ohio Clinical Research Associates Inc., Mayfield Heights, Ohio, USA), Alan Koterba, MD (Allergy Associated of the Palm Beaches, Palm Beach, Florida, USA); Rebecca Avila, MS (ADMA Biologics, Boca Raton, Florida, USA); Miranda Anaya, PharmD (ADMA Biologics, Boca Raton, Florida, USA); Marie-Chantale Simard, PhD (ADMA Biologics, Boca Raton, Florida, USA); Wei Du, PhD (Clinical Statistics Consulting, Blue Bell, Pennsylvania, USA).

^‡‡^ Department of Pediatrics, Division of Allergy and Immunology, Johns Hopkins University, Baltimore, Maryland, USA

^§§^ Cancer and Blood Disorders Institute and Institute for Clinical and Translational Research, Johns Hopkins All Children’s Hospital, St. Petersburg, Florida, USA

^‖‖^ Institute for Clinical and Translational Research, Johns Hopkins All Children’s Hospital, St. Petersburg, Florida, USA

**Corresponding author:**

Jennifer Leiding, MD

Johns Hopkins All Children’s Hospital

Research and Education Building

600 Fifth Street South, Suite 3200, St. Petersburg, FL 33701.

Telephone number: 727-767-3674

Fax: 727-767-6949

Email: jleidin1@jhmi.edu

## Supplementary Methods

### Recruitment

The recruitment target was 18 evaluable participants (defined as those completing pharmacokinetic sampling), including six in each of the following age groups: 2 to <6 years, 6 to <12 years, and 12 to 16 years. Participants were required to have a documented diagnosis of PID (including hypogammaglobulinemia or agammaglobulinemia), have received intravenous immune globulin infusions every 3 or 4 weeks at a steady dose (maintained at ±25% of the mean dose) for ≥3 months prior to study entry, and maintain an immune globulin G (IgG) trough level of at least 500 mg/dL. Participants could not have known intolerance or allergic reaction to immune globulins, absolute immune globulin A deficiency or known antibodies to immune globulin A, any medical condition or finding precluding participation (including medical conditions known to cause secondary immune deficiency), a serious bacterial infection (SBI) within the last 3 months, a current active infection with antibiotic use, a history of thrombotic events within 6 months before the first IVIG 10% dose (i.e., prior to study drug), pre-existing risk factors for thrombotic events, a diagnosis of hepatitis B or hepatitis C, a positive human immunodeficiency virus test, or a screening laboratory value of ˃2.5 times the age-specific upper limit of normal for alanine aminotransferase, aspartate aminotransferase, lactate dehydrogenase, blood urea nitrogen, or serum creatinine.

### Ethics

This study was conducted in accordance with the revised Declaration of Helsinki (2013), International Conference on Harmonization E6/Good Clinical Practice Guidelines for the conduct of Clinical Trial Investigations (1996) and in compliance with the requirements of the clinical trial regulatory guidelines and regulations. Written informed consent was obtained from participants’ parent/legal guardian indicating that they understood the study purpose and procedures and were willing to participate. All sites were activated following institutional review board approval.

### Procedures

This study took place from December 2016 to June 2021 at six sites in the US. In line with the dose and schedule used in the pivotal study, participants received IVIG 10% for 5 months at the dose of 300–800 mg/kg every 3 or 4 weeks; this was the same dose/schedule as their previous intravenous immune globulin maintenance therapy. Dose adjustments were permitted during the study to maintain trough total IgG concentrations at >500 mg/dL, but dose increases >800 mg/kg required approval by the sponsor’s medical director or designee. Per recommended infusion rates for the study drug, IVIG 10% was infused at 0.5 mg/kg/min for 10 min and the rate was increased by 0.8 mg/kg/min every 20 min to a maximum of 6 mg/kg/min if tolerated. Blood samples for assessing trough levels of total IgG were taken prior to each infusion (infusions 1–7 for the 3-week regimen group or infusions 1–5 for the 4-week regimen group).

### Outcomes

Safety parameters were the primary objective and included the incidence of temporally-associated adverse events (defined as adverse events [AEs] occurring during or within 1, 24, or 72 h of completion of an infusion), incidence of serious adverse events (SAEs), incidence of treatment-emergent adverse events (TEAEs), total number and incidence of adverse reactions and suspected adverse reactions, incidence of adverse infusion-related reactions, and incidence of infusion site reactions.

Efficacy and pharmacokinetics were secondary objectives. Incidence of acute SBIs (acute bacterial pneumonia, bacteremia/sepsis, bacterial meningitis, visceral abscess, or osteomyelitis/septic arthritis) was the pre-specified primary efficacy endpoint. Other efficacy parameters included all infections of any kind (serious and non-serious), time to first infection, time to resolution of infections, number of days of antibiotic treatment, number of school or work days missed due to infections and their treatment, number of infections, days of fever (≥38°C/100.4°F), and days of hospitalizations due to infections.

Pharmacokinetics evaluation included serum concentrations of total IgG and subclasses IgG1, IgG2, IgG3, and IgG4, measured using validated enzyme-linked immunosorbent assays. The following parameters were calculated for total IgG: maximum serum concentration, time to maximum serum concentration, minimum serum concentration, elimination rate constant, elimination half-life, area under the concentration-time curve to the last concentration ≥ lower level of quantification, area under the curve over the dosing interval, serum clearance, and volume of distribution.

### Data Analysis

Safety data and secondary efficacy outcomes were summarized using descriptive statistics. Percentages reported may not add up to 100 due to rounding. All participants who received at least one dose of IVIG 10% and had at least one post-dose pharmacokinetics assessment, including one blood pressure recording, and one pharmacokinetic laboratory draw, were included in the pharmacokinetics analysis set. Data were analyzed using SAS^®^ software (SAS Institute, North Carolina) Version 9.4.

**
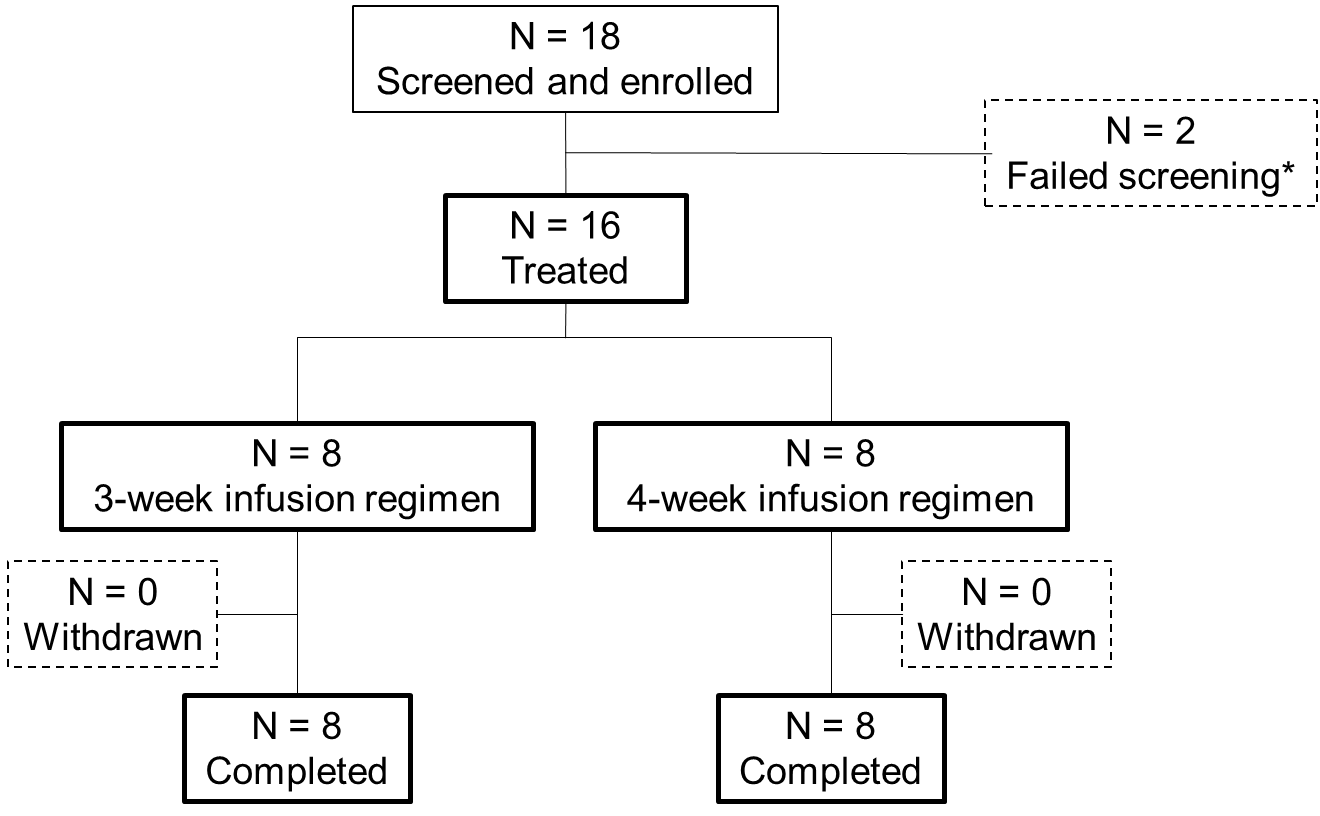
**

**Figure S1** – Study flowchart

*One participant was excluded due to an active infection and having received antibiotic therapy for its treatment at the time of screening. Another (female) participant was excluded due to because of oral contraceptive use

**Table S1** – Demographics and clinical characteristics at baseline

|  | 3-week regimen (N=8*) | 4-week regimen (N=8*) | Total (N=16*) |
| --- | --- | --- | --- |
| Age, years |  |  |  |
| Mean (SD) [range] | 11.0 (5.2) [3–16] | 9.5 (3.1) [5–13] | 10.3 (4.2) [3–16] |
| Age range, n (%) |  |  |  |
| 2–<6 years | 2 (25) | 1 (13) | 3 (19) |
| 6–<12 years | 1 (13) | 4 (50) | 5 (31) |
| 12–16 years | 5 (63) | 3 (38) | 8 (50) |
| Sex, n (%) |  |  |  |
| Male | 8 (100) | 8 (100) | 16 (100) |
| Race, n (%) |  |  |  |
| White | 7 (88) | 6 (75) | 13 (81) |
| Black or African American | 0 | 1 (13) | 1 (6) |
| American Indian or Alaska Native | 1 (13) | 0 | 1 (6) |
| Other | 0 | 1 (13) | 1 (6) |
| Ethnicity, n (%) |  |  |  |
| Not Hispanic/Latino/Spanish origin | 6 (75) | 7 (88) | 13 (81) |
| Hispanic/Latino/Spanish origin | 2 (25) | 1 (13) | 3 (19) |
| Weight, kg |  |  |  |
| Mean (SD) | 50.1 (32.7) | 37.4 (17.2) | 43.7 (26.1) |
| PID diagnosis, n (%) |  |  |  |
| Hypogammaglobulinemia | 3 (38) | 5 (63) | 8 (50) |
| Common variable immunodeficiency | 3 (38) | 1 (13) | 4 (25) |
| Combined immunodeficiency | 2 (25) | 0 | 2 (13) |
| Bruton's agammaglobulinemia | 0 | 1 (13) | 1 (6) |
| Selective polysaccharide antibody deficiency | 0 | 1 (13) | 1 (6) |
| Time since PID diagnosis, years^†^ |  |  |  |
| Mean (SD) [range] | 3.9 (2.2) [1.6–7.1] | 4.7 (4.2) [0.9–10.8] | 4.3 (3.3) [0.9–10.8] |
| IgG trough level, mg/dL |  |  |  |
| At screening, mean (SD) | 1016.4 (181.3) | 821.4 (183.1) | 918.9 (202.8) |
| At pre-infusion 1, mean (SD) | 964.3 (127.6) | 760.3 (182.7) | 862.3 (185.1) |

*For time since PID diagnosis, N=7 for each regimen (N=14 total)

^†^Calculated as (date of informed consent – PID diagnosis date + 1)/365.25

IgG = immune globulin G; PID = primary immune deficiency; SD = standard deviation

**Table S2** – Summary of TEAEs by severity

|  | 3-week regimen (N=8) | 4-week regimen (N=8) | Total  (N=16) |
| --- | --- | --- | --- |
| Participants with ≥1 TEAE, n (%) | 8 (100) | 5 (63) | 13 (81) |
| Mild | 2 (25) | 2 (25) | 4 (25) |
| Moderate | 5 (63) | 3 (38) | 8 (50) |
| Severe | 1 (13) | 0 | 1 (6) |
| Total number of TEAEs, n (%) | 62 | 12 | 74 |
| Mild | 30 (48) | 2 (17) | 32 (43) |
| Moderate | 31 (50) | 10 (83) | 41 (55) |
| Severe | 1 (2) | 0 | 1 (1) |

TEAE = treatment-emergent adverse event

**Table S3** – Incidence of acute SBIs

|  | 3-week regimen (N=8) | 4-week regimen (N=8) | Total (N=16) |
| --- | --- | --- | --- |
| Acute SBI episodes, n (%) | 0 | 0 | 0 |
| Acute SBI episodes per person-years* |  |  |  |
| Mean (SD) [range] | 0 (0) [–] | 0 (0) [–] | 0 (0) [–] |
| Length of observation, days |  |  |  |
| Mean (SD) [range] | 158.1 (18.6) [134–196] | 145.9 (13.1) [136–177] | 152.0 (16.8) [134–196] |
| Days to first acute SBI episode | – | – | – |

*Calculated per participant as 365 × *n/d*, where *n* is the number of episodes and *d* is the length of observation (days from first infusion until acute SBI onset, death, or date of last study visit)

SBI = serious bacterial infection; SD = standard deviation

**Table S4** – Other secondary efficacy endpoints

|  | 3-week regimen (N=8) | 4-week regimen (N=8) | Total (N=16) |
| --- | --- | --- | --- |
| Infections (serious and non-serious) on study | 16 | 1 | 17 |
| Infections (serious and non-serious) per participant | N=8 | N=8 | N=16 |
| Mean (SD) [range] | 2.0 (2.1) [0–6] | 0.1 (0.4) [0–1] | 1.1 (1.8) [0–6] |
| Serious infections on study | 0 | 0 | 0 |
| Non-serious infections on study | 16 | 1 | 17 |
| Non-serious infections per participant | N=8 | N=8 | N=16 |
| Mean (SD) [range] | 2.0 (2.1) [0–6] | 0.1 (0.4) [0–1] | 1.1 (1.8) [0–6] |
| Days to first infection per participant***** | N=6 | N=1 | N=7 |
| Mean (SD) [range] | 69.3 (48.9) [14–123] | 75.0 (–) [75–75] | 70.1 (44.7) [14–123] |
| Days to resolution of infection, mean (SD) [range]^†^ | 15.3 (11.8) [2–48] | 3.0 (–) [3–3] | 14.5 (11.8) [2–48] |
| Duration of infections on study, days | 172 | 3 | 175 |
| Duration of infections per participant, days^‡^ | N=8 | N=8 | N=16 |
| Mean (SD) [range] | 21.5 (29.6) [0–78] | 0.4 (1.1) [0–3] | 10.9 (23.0) [0–78] |
| Duration of antibiotic treatments on study, days | 64 | 35 | 99 |
| Duration of antibiotic treatments per participant, days | N=5 | N=1 | N=6 |
| Mean (SD) [range] | 12.8 (11.3) [1–31] | 35.0 (–) [35–35] | 16.5 (13.6) [1–35] |
| Days missed school/work on study | 9 | 0 | 9 |
| Days missed school/work per participant | N=1 | N=0 | N=1 |
| Mean (SD) [range] | 9 (–) [–] | 0 (0) [–] | 9 (–) [–] |
| Days hospitalized on study | 0 | 0 | 0 |
| Days with fever ≥38ºC on study^§^ | 0 | 0 | 0 |

*****Earliest start date – first infusion date + 1

^†^Per infection; end date – start date + 1

^‡^Per participant; if >1 infection, their sum was used

^§^Two events of fever occurred but were not included in analyses due to body temperature not reported

SD = standard deviation

**Table S5** – Individual participant trough serum concentrations of total IgG and IgG subclasses

| Infusion regimen | Age group, years | Participant | Dose*****, mg/kg | Serum concentration (mg/dL) | | | | | | | | | |
| --- | --- | --- | --- | --- | --- | --- | --- | --- | --- | --- | --- | --- | --- |
|  |  |  |  | IgG | | IgG1 | | IgG2 | | IgG3 | | IgG4 | |
|  |  |  |  | Visit 1 | Visit 5/7^†^ | Visit 1 | Visit 5/7^†^ | Visit 1 | Visit 5/7^†^ | Visit 1 | Visit 5/7^†^ | Visit 1 | Visit 5/7^†^ |
| 3-week | 2–<6 | 1 | 1,104.0 | – | 1,310 | – | 786 | – | 470 | – | 56 | – | 21 |
|  | 12–16 | 2 | 489.0 | 1,180 | 1,080 | 763 | 715 | 355 | 352 | 43 | 34 | 24 | 24 |
|  | 12–16 | 3 | 635.0 | 1,120 | 1,010 | 649 | 588 | 425 | 351 | 62 | 68 | 22 | 14 |
| 4-week | 2–<6 | 4 | 537.6 | 606 | 623 | 274 | 355 | 262 | 248 | 8 | 12 | 12 | 9 |
|  | 6–<12 | 5 | 672.8 | – | 641 | – | 390 | – | 277 | – | 20 | – | 9 |
|  | 6–<12 | 6 | 510.2 | 813 | 849 | 541 | 540 | 149 | 178 | 100 | 116 | 8 | 9 |
|  | 6–<12 | 7 | 400.0 | – | 853 | – | 449 | – | 260 | – | 59 | – | 19 |
|  | 12–16 | 8 | 604.0 | 971 | 691 | 562 | 410 | 338 | 271 | 27 | 18 | 12 | 8 |
|  | 12–16 | 9 | 356.1 | 624 | 752 | 328 | 370 | 241 | 239 | 53 | 49 | 8 | 10 |
|  | 12–16 | 10 | 463.0 | 1,130 | 1,050 | 640 | 586 | 337 | 352 | 22 | 24 | 36 | 33 |

*****Dose for final infusion

^†^Measurement at visit 7 (3-week regimen group) or 5 (4-week regimen group)

IgG = immunoglobulin G

**Table S6** – Individual participant total IgG pharmacokinetics parameters

| Regimen***** | Age group | Participant | Dose, mg/kg | Weight, kg^†^ | C_min_, µg/dL | C_max_, µg/dL | T_max_, h | AUC_0–t_,  h·µg/dL | AUC_0–tau_, h·µg/dL^‡^ | λ_Z_, h^–1^ | t½, h^§^ | CL, mL/h/kg |
| --- | --- | --- | --- | --- | --- | --- | --- | --- | --- | --- | --- | --- |
| 3-week | 2–<6 | 1 | 1,104.0 | 16.3 | 1,250.0 | 2,680.0 | 2.3 | 816,700 | 816,700 | 0.00123 | 564.9 | 135.2 |
|  | 12–16 | 2 | 489.0 | 59.0 | 1,080.0 | 2,040.0 | 6.5 | 540,778 | – | – | – | – |
|  | 12–16 | 3 | 635.0 | 61.2 | 1,010.0 | 3,130.0 | 2.5 | 717,848 | – | – | – | – |
| 4-week | 2–<6 | 4 | 537.6 | 18.6 | 623.0 | 1,540.0 | 7.6 | 661,046 | 661,046 | 0.00080 | 871.3 | 81.3 |
|  | 6–<12 | 5 | 672.8 | 31.1 | 641.0 | 2,780.0 | 2.8 | 424,178 | – | 0.00177 | 391.6 | – |
|  | 6–<12 | 6 | 510.2 | 19.7 | 849.0 | 1,570.0 | 3.1 | 702,513 | 702,513 | 0.00053 | 1,316.1 | 72.6 |
|  | 6–<12 | 7 | 400.0 | 28.6 | 853.0 | 1,440.0 | 2.1 | 850,949 | 850,949 | – | – | 47.0 |
|  | 12–16 | 8 | 604.0 | 32.2 | 691.0 | 1,510.0 | 1.5 | 742,700 | 742,700 | – | – | 81.3 |
|  | 12–16 | 9 | 356.1 | 67.1 | 752.0 | 1,600.0 | 7.8 | 677,384 | 677,384 | – | – | 52.6 |
|  | 12–16 | 10 | 463.0 | 54.0 | 1,050.0 | 2,020.0 | 3.2 | 45,113 | – | – | – | – |

*****Measurements at visit 7 (3-week regimen group) or 5 (4-week regimen group)

^†^Weight at screening visit

^‡^AUC_(0–tau)_ only estimated if the final time is approximately the dosing interval

^§^t½ > dosing interval and reported for informational purposes only

AUC_0–t_ = area under the serum concentration-time curve to the last concentration equal to or above the lower level of quantification; AUC_0–tau_ = area under the serum concentration-time curve over the dosing interval; CL = serum clearance; C_max_ = maximum serum concentration; C_min_ = minimum serum concentration; t½ = elimination half-life; T_max_ = time of maximum serum concentration; λ_z_ = elimination rate constant
